# Electrical Activity of the Diaphragm in a Small Cohort of Preterm Infants on Noninvasive Neurally Adjusted Ventilatory Assist and Continuous Positive Airway Pressure: A Prospective Comparative Pilot Study

**DOI:** 10.7759/cureus.6291

**Published:** 2019-12-04

**Authors:** Arpit Gupta, Rishi Lumba, Sean Bailey, Sourabh Verma, Uday Patil, Pradeep Mally

**Affiliations:** 1 Neonatology, Metropolitan Hospital Center, New York, USA; 2 Pediatrics/Neonatal Perinatal Medicine, New York University School of Medicine, New York, USA; 3 Pediatrics, New York University School of Medicine, New York, USA; 4 Pediatrics/Neonatal Perinatal Medicine, Icahn School of Medicine at Mt. Sinai & New York City H + H / Elmhurst, Elmhurst, USA

**Keywords:** noninvasive ventilation, neurally adjusted ventilatory assist, preterm, electrical activity of the diaphragm, biphasic, cpap

## Abstract

Objective: To compare the electrical activity of the diaphragm (Edi) of premature infants placed on continuous positive airway pressure (CPAP) with the Edi of premature infants placed on noninvasive neurally adjusted ventilatory assist (NIV NAVA). The secondary aim was to evaluate the feasibility of the use of NIV NAVA mode in the busy tertiary neonatal unit.

Study Design: This was a prospective crossover pilot study of premature infants requiring noninvasive respiratory support. Infants were randomized to initially receive either CPAP/biphasic (group 1) or NIV NAVA (group 2) and were then crossed over to the alternate group. Continuous Edi signals were recorded for 24 h, with 12 h each on CPAP/biphasic, and NIV NAVA along with other clinical and respiratory parameters.

Results: Ten infants with a mean gestation age of 29 weeks (range 25-34 weeks) were enrolled, with a total cumulative Edi monitoring of 240 h. The average Edi peak on the biphasic/CPAP group (15.6 ± 7 mcV) was significantly higher (P < 0.005), compared to the Edi Peak on the NIV NAVA group (10.8 ± 3.3 mcV). The Edi min values were 3.23 ± 1.1 mcV, and 3.07 ± 0.5 mcV on CPAP/biphasic and NIV NAVA (P = 0.69) respectively. There were no significant differences in other clinical parameters between the two groups. No major adverse events were recorded during Edi catheter monitoring.

Conclusion: The Edi peak values were significantly lower in NIV NAVA mode compared to CPAP/biphasic mode. The Edi catheter and NIV NAVA may also be used safely in premature infants.

## Introduction

Many infants both preterm and term require noninvasive respiratory support in the neonatal intensive care unit. Noninvasive support can be provided using different modalities such as high flow nasal cannula (HFNC), continuous positive airway pressure (CPAP), biphasic, nasal noninvasive intermittent positive pressure ventilation (NIPPV), noninvasive neurally adjusted ventilatory assist (NIV NAVA) [1-2], etc.

Nasal CPAP and nasal biphasic mode are one of the widely used modes of noninvasive respiratory ventilation in neonates. CPAP is a constant single level of positive pressure to the infant’s airway, facilitating the restoration of functional residual capacity (FRC) [3-4]. Biphasic, on the other hand, is a modality used for babies that require more respiratory support than CPAP can provide [5]. Biphasic provides alternating levels of high and low positive pressures (PEEP). In biphasic mode, respiratory rate (RR), inspiratory time (Ti), and peak inspiratory pressures (PIPs) are set and not synchronized with the patients’ breathing efforts [6]. Since in both CPAP and biphasic mode, positive pressure is delivered using facial or nasal interface, the delivery of PEEP can be greatly affected by leakage [7-10]. 

 NIV NAVA, on the other hand, is a neurally controlled noninvasive ventilation mode. It provides synchronized assist independent of conventional pneumatic sensors and leakage associated with patient interface [11-13]. NIV NAVA manages asynchrony, as the mode does not rely on a pneumatic signal and is not affected by auto-PEEP [11, 13]. NAVA utilizes a special nasogastric catheter called an electrical activity of a diagram (Edi) catheter which has embedded electrodes and is positioned at the level of the diaphragm. It measures the amplitude, duration, and frequency of the Edi signals, and uses these signals to trigger and cycle-off the ventilator as well as to adapt to the amount of pressure delivered [14].

The Edi peak represents the neural inspiratory effort and is the amount of electrical activity needed to generate the required tidal volume [11, 14]. It refers to the amplitude of electrical activity associated with respiratory effort, and therefore diaphragmatic workload. As the work of breathing increases, the neural signal sent to the diaphragm causes an increased Edi peak in order to maintain effective ventilation. As the work of breathing decreases, Edi will return to baseline, indicating the decrease in diaphragmatic workload [15]. The Edi min (minimum) represents the tonicity of the diaphragm at rest and helps to maintain regulate end-expiratory lung volume and prevents decruitment [11, 14].

 The NAVA level is a proportionality factor that converts the Edi signal into a proportional pressure and allows the ventilator to be in complete synchrony with the patient, who determines its own peak pressure, respiratory rate, inspiratory and expiratory times on a breath-by-breath basis [14, 16]. Increasing the NAVA level initially increases the respiratory support (peak pressure) while maintaining its Edi until the individual reaches its breakpoint which represents optimal respiratory muscle off-loading [17]. Further increase in NAVA level causes over-supporting thereby causing small Edi signals (<5 mcV). Inadequate support, on the other hand, leads to an increase in Edi signals (>20 mcV) [17]. As shown by different authors, the optimal Edi peak in a well-supported neonate is usually between 5 and 16 mcV and Edi min is usually less than 5 mcV [18-20]. These target Edi peaks represent optimal unloading of respiratory muscles including the diaphragm and generating desired tidal volume.

NIV-NAVA is a relatively newer mode of noninvasive ventilation as compared to the CPAP/biphasic mode, and its use has been approved by the Food and Drug Administration (FDA) in infants. It has been shown as a safer and effective mode of noninvasive ventilation in premature and term infants [15]. As NAVA is neurally driven it has been shown [14, 20-21] to improve synchrony between patient and machine.

The support on NIV NAVA is proportional to the patient’s self-need and is based on their Edi feedback, whereas the support in CPAP/biphasic mode is not dynamic but is preset. Our primary aim was to compare the Edi on NIV NAVA mode to the Edi on nasal CPAP/biphasic mode in premature infants and evaluate whether there was any significant difference in the Edi activity on these noninvasive modes.

The secondary aims were to monitor the efficacy and feasibility of noninvasive NAVA ventilation in a busy clinical setting and to monitor any adverse events related to the Edi catheter placement in a preterm population. The data of the use of NIV NAVA in the preterm population is still limited with most studies showing no significant complications compared to other NIV modes. This study will add up to the limited existing literature of the use of NIV NAVA in this venerable population.

## Materials and methods

This was a prospective, randomized, cross-over physiologic study comparing CPAP/biphasic to NIV NAVA in a tertiary level III unit between the period of July 2014 and June 2015. The primary aim of this study was to compare the Edi signals in neonates placed on either biphasic/CPAP or noninvasive NAVA (NIV NAVA) mode. The secondary aims were to evaluate the feasibility of the use of Edi catheter in the neonates and the overall feasibility of implementing NIV NAVA in a busy clinical setting.

Preterm infants ranging from 26 weeks to 34 weeks gestation requiring noninvasive ventilatory support in neonatal intensive care unit (NICU) were enrolled. There were two arms of patient enrollment. The first arm consisted of neonates admitted to the NICU and requiring noninvasive support, and the second arm consisted of neonates who were initially intubated, but extubated to noninvasive support. The infants were deemed to be ready for extubation if they were hemodynamically stable, and requiring FiO2 < 40%, peak pressure < 14 cmH2O, MAP < 8 cmH2O, and having spontaneous breathing efforts. The infants with congenital anomalies or grade II or higher interventricular hemorrhage (IVH) were excluded from the study. The study was approved by the institutional review board, and all parents gave informed consent. 

Neonates in the study were assigned alternatively by the primary team to either of the two groups CPAP/biphasic or NIV NAVA group with the first newborn enrolled in the CPAP group. This was done to minimize selection bias. The patients stayed on the selected mode for 12 h followed with crossover to the alternate mode. CPAP/biphasic mode was provided with infant flow SIPAP system (CareFusion, Yorba Linda, CA), whereas noninvasive NAVA mode was provided with Servo-I ventilators (MAQUET, Solna, Sweden) equipped with version 7.0 software. Edi catheter (MAQUET, Solna, Sweden) was placed in all neonates instead of a regular nasogastric tube to continuously monitor the Edi signals. The size of the catheter used was in accordance with the manufacturer guidelines. The level of support on either CPAP or biphasic was decided by the primary medical team with a lower PEEP of 5 cmH2O, upper PEEP of 3 cmH2O above baseline, and frequency of 15-20 cycles per minute. On noninvasive NAVA, the NAVA level was initially set at 1 cmH2O/mcV, and then titrated based on the clinical parameters, PIPs, and Edi signals trends; the Edi trigger was set at 0.5 mcV. Targeted SpO2 was in accordance with unit protocol (88%-92% for sub-1000 g, and 91%-94% for other preterm infants under 34 weeks). The currently acceptable oxygen saturation is now changed to 91%-94% in sub-1000 g. The backup ventilation rate on NIV NAVA mode was set to 30 per minute.

All infants were continuously monitored and would be de-enrolled from the study if any of the following problems developed: increase in oxygen demand by 10% above the baseline, respiratory rate > 80/min, heart rate > 180/min, hemodynamic instability or increase in overall apneic episodes. All infants were on caffeine during the study period. All infants were also monitored for feeding intolerance, abdominal distension, bilious emesis, and pneumothorax during the study.

A minimum of 24 h continuous data monitoring, 12 h each on CPAP/biphasic mode and 12 h on NIV NAVA mode was done, using the NAVA module of Servo-I software. Subsequent data collected was then uploaded into a computer for analysis using the Maquet Servo-I download card. Information collected was then divided into 30 min intervals, with the first 30 min of data not being used to minimize variations and to let the infants stabilize. Average values and the ranges were then calculated and imported into Microsoft Excel sheet for analysis. Average values for PIPs, and mean airway pressure were also collected on noninvasive NAVA mode. Clinical data including heart rate, respiratory rate, mean blood pressure, and oxygen saturation were continuously monitored using the unit standard monitors.

**Figure 1:**
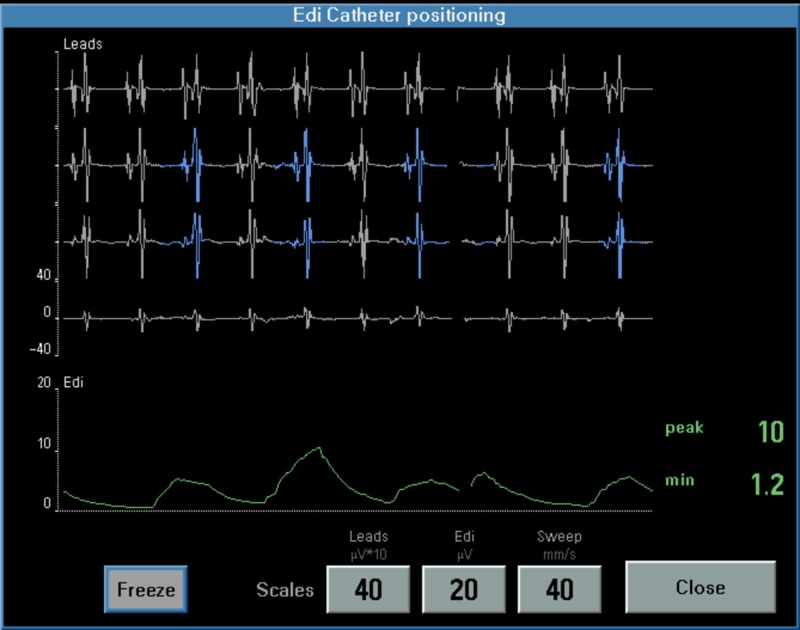
Position of Edi catheter with blue signals in 2nd and 3rd tracings and no ‘P’ wave in 4th tracing.

Edi catheters were placed by the bedside nurse and the position was regularly monitored by respiratory therapists. Position was considered optimal if the second and third electrocardiogram tracings displayed a blue color and the overall amplitude of the tracing reduced from the first tracing to the fourth tracing with no ‘p’ wave in fourth tracing [13] (Figure 1). Neonates were also monitored for any catheter-related adverse events or any change in routine clinical status.

Data analysis

 All results were expressed in terms of mean ± SD. Statistical analyses were performed using SPSS v. 21.0 (Chicago, IL, USA) software. Differences in continuous variables between study groups were assessed by using Student’s t-test. A P value < 0.05 was considered significant for all statistical analyses.

## Results

A total of 10 neonates, five males, and five females were each enrolled in the study. The average gestation at birth was 29 weeks with a range between 25 2/7 weeks and 33 4/7 weeks (Table 1). The mean gestational age at the time of enrollment was 30 weeks with a range between 26 weeks and 34 weeks. The average birth weight was 1265 ± 403 g with a range of 600-1800 g. The mean age at the time of enrollment was 6.3 ± 7.7 (1-26) days. Some 90% of neonates had appropriate weight for their gestation and only one infant was small for gestation. Of the 10 neonates, 60% was born to Caucasian mothers and 40% was born to Hispanic mothers. 70% of neonates were exposed to the full course of prenatal steroids. The mode of delivery was C-section in 90% of the neonates. Out of nine, seven neonates were delivered prematurely for maternal indication and two neonates were delivered for nonreassuring intrapartum fetal tracings. One neonate was delivered by vaginal delivery for preterm labor. All neonates had respiratory distress syndrome (RDS) at birth, and two neonates (20%) received surfactant. Some 50% of neonates were intubated at some point before the enrollment in the study. All of the neonates were on caffeine during noninvasive ventilation. Some 30% of neonates had patent ductus arteriosus (PDA) and were treated with indomethacin. Overall, one infant (10%) developed chronic lung disease. None of the neonates developed a pneumothorax, intraventricular hemorrhage (IVH), and necrotizing enterocolitis (NEC).

**Table 1:** Subjects demographics showing their gestation age (GA), birth weight (BW), corrected gestation age (CGA), mode of delivery (MOD), delivery indication, race, prenatal steroids, Apgar score at 1 and 5 min, and maternal prenatal antibiotics. NSVD – normal spontaneous vaginal delivery, CS – cesarean section, PEC – pre-eclampsia, P. previa – placenta previa, PTL – preterm labor, F distress – fetal distress, C – Caucasian, H – Hispanic, M – male, F - female.

Subjects	GA (weeks)	Sex	BW (g)	CGA	MOD	Indication	Race	Prenatal steroids	Apgar	Prenatal antibiotics
1	27 ^4/7^	F	930	29	CS	PEC	C	Yes	4, 6	Yes
2	33 ^1/7^	F	1750	33	CS	P. previa	C	Yes	8, 9	No
3	30 ^1/7^	M	1520	30	CS	PTL	H	Yes	7, 8	Yes
4	33 ^4/7^	M	1800	33	NSVD	PTL	C	No	8, 9	Yes
5	29 ^3/7^	F	1070	30	CS	PEC	C	Yes	5, 7	No
6	25 ^2/7^	M	850	29	CS	PTL	C	Yes	6, 8	Yes
7	25 ^3/7^	M	600	27	CS	F distress	H	Yes	5, 7	Yes
8	30 ^3/7^	F	1150	31	CS	PEC	H	Yes	6, 8	No
9	28 ^2/7^	M	1450	29	CS	PTL	H	Yes	6, 7	Yes
10	31 ^3/7^	F	1530	32	CS	F distress	C	yes	4, 7	No

A total of ten crossovers between the two groups occurred, with 12 h of monitoring each on biphasic/CPAP, and noninvasive NAVA during the study, giving a cumulative time of 240 h of data. In the CPAP/biphasic group, six infants received biphasic support and four neonates received CPAP support.

**Figure 2:**
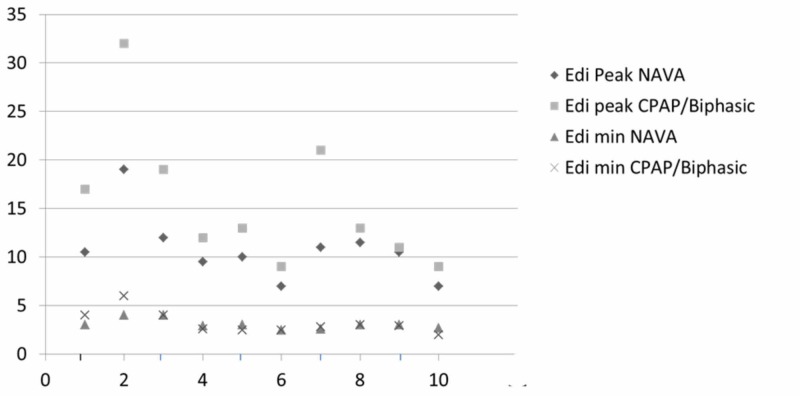
Edi peak and min values of 10 subjects on NIV NAVA and CPAP/biphasic modes. On X axis – number of newborns from 1 to 10. On Y axis – electrical activity (Edi) of diaphragm in microvolts (mcV). CPAP – continuous positive airway pressure, NIV NAVA – noninvasive neurally adjusted ventilatory assist

 The overall Edi peak on biphasic/CPAP mode was 15.6 ± 7 mcV, and on NIV NAVA was 10.8 ± 3.3 mcV (Table 2). The difference in Edi peak between the two groups was statistically significant with a P-value of 0.005. The overall Edi min on CPAP/biphasic mode was 3.23 ± 1.1 mcV, and on NIV NAVA was 3.07 ± 0.5 mcV, with no statistical difference in EDI min detected between groups. The Edi peak range on CPAP/biphasic was 9-32 mcV, and on NIV NAVA was 7-19 mcV. The Edi min range was 2-6 mcV on CPAP/biphasic, and 2.5-4 mcV on NIV NAVA (Figure 2).

**Table 2:** Mean values of Edi signals, HR, RR, MBP, SpO2, and FiO2 on CPAP/biphasic and NIV NAVA mode. CPAP – continuous positive airway pressure, NIV NAVA – noninvasive neurally adjusted ventilatory assist, HR – heart rate, RR – respiratory rate, SpO2 – oxygen saturation, MBP – mean blood pressure, FiO2 – inspired oxygen concentration, mcV - microvolts.

N = 10	Mean values on CPAP/Biphasic	Mean values on NAVA	P values
Edi peak (mcV)	15.6 ± 7	10.8 ± 3.3	0.005
Edi min (mcV)	3.23 ± 1	3.07 ± 0.5	0.693
HR (rate/min)	146.9	150.6	0.428
RR (rate/min)	49.5	46.2	0.511
MBP (mm/Hg)	43.7	44.2	0.851
SpO_2_	97.1	97.2	0.913
FiO_2_ (%)	23.3	23.8	0.737

Table 2 shows the average values and compares the heart rate (HR), respiratory rate (RR), mean blood pressure (MBP), oxygen saturation (SpO2), and inspired oxygen saturation (FiO2) on two different cross-over modes. There was no significant difference between these parameters. The average PIP and MAP on noninvasive NAVA were 15 ± 4 cmH2O and 6.5 ± 1.3 cmH2O respectively. The average MAP was 6.3 (8-6) cmH2O in biphasic infants and 5 cmH2O in CPAP infants. The average NAVA level was 1.60 ± 0.2 cmH2O/mcV. All infants remained stable during the study. 

All 10 neonates tolerated Edi catheter placement and had no adverse events during the study. Edi catheters signaling on the ventilator screen during the study was consistent with no major disruptions. No adverse effects, such as feeding intolerance, significant abdominal distension, hemodynamic instability, and pneumothorax were reported during the course of the monitoring.

## Discussion

There is a growing but still limited data on the use and safety of NIV NAVA in premature infants. Most of the previously reported studies like our current study had small patient population ranging from 5 to 15 [18, 20, 22]. The NIV NAVA mode as used in our study seems to be effective as well as safe compared to the other existing noninvasive modes in premature infants. 

It is often difficult to compare any noninvasive mode head to head in a short period. Most of the existing noninvasive methods of ventilation used in neonates have been found to be effective and safe. However, there is a theoretical advantage of NIV NAVA over other existing modes. NIV NAVA gives us constant and live feedback in terms of Edi peak and Edi min values. It can also assist in minimizing asynchrony events like ineffective efforts and autotriggering as demonstrated by Lee et al. [23]. Edi waveform is an estimate of the patient’s own neuronal drive and is independent of any leaks giving information about lung stretch, respiratory muscle loading, and other inputs [17]. The utility of Edi monitoring while on respiratory support can be compared to the utility of end-tidal CO2 or transcutaneous CO2 monitoring in the intensive unit [24]. Since the majority of premature infants needing NIV require feeding catheters for either feeding or gastric decompression, Edi catheter tracings can be considered as an additional bedside clinical tool to monitor the effectiveness of the noninvasive support. 

Edi peak in neonates represents tidal inspiratory pressure and varies from breath to breath whereas Edi min represents the force required to prevent lung decruitment and thus maintain FRC. There are enough clinical studies reporting the optimal Edi peak and Edi values on NIV NAVA in both term and preterm infants. In a study done on nonintubated preterm infants by Stein et al. [25], the average Edi peak was 10.8 ± 3.7 mcV and Edi min was 2.8 ± 1.1 mcV. The infants in their study were monitored on HFNC, nasal cannula, and room air with an average birth weight of 1220 (628-2520 g). The authors reported no change in Edi values over different gestational ages. In a different study done on term neonates, Stein et al. reported mean Edi peak of 11 ± 5 μV and mean Edi minimum of 3 ± 2 μV [18].

In a cross-over trial, Lee et al. [23] compared NIV NAVA with NIV pressure support (PS) in 15 preterm infants born under 32 weeks. The mean maximum Edi or Edi peak was 12.6 ± 6.3 in NIV NAVA compared to 16.6 ± 8.7 mcV (P 0.003) in NIV PS mode. They titrated the NAVA level in NIV NAVA mode to maintain the same transcutaneous PCO2 as on NIV PS mode. They reported significantly lower ventilator trigger delay (Td), ventilator inspiratory time (Ti ventilator), and inspiratory excess in time (Ti excess) on NIV NAVA compared to NIV PS mode demonstrating improved synchrony on NIV NAVA leading to lower maximum (peak) Edi and lower swing Edi (proportional to delta P). This may represent improved compliance and optimal respiratory muscle unloading. Houtekie et al. [21] in a similar prospective cross-over study reported decreased work of breathing on NIV NAVA as compared to nasal CPAP. In their study, they randomized infants after extubation from cardiac surgery into NIV NAVA group and CPAP group and studied for 30 min each in both groups. All their children were ventilated using the same interface. They reported Edi peak of 10.5 ± 5.6 mcV in NIV NAVA mode as compared to 16.9 ± 7.6 mcV in CPAP mode with P < 0.001. In a recently reported study, Yonehara et al. [26] compared NIV NAVA to NIPPV in preterm born before 30 weeks postextubation. They reported no difference in the treatment (primary extubation) failure between two groups with no significant difference in adverse events.

The mean NAVA level in our study was 1.5 ± 0.2 which was comparable to that reported by different authors in preterm studies. The mean PIP and mean RR were 15 ± 4 cmH20 and 46 ± 9 breaths per minute respectively on NIV NAVA. All these respiratory variables were consistent with the current understanding of respiratory physiology in newborns as described by Stein et al. recently [27].

Although the work of breathing was not measured in this study, the lower Edi peak on NIV NAVA may suggest improved synchronization as reported in earlier studies. In NIV NAVA, they were able to get appropriate support based on clinical parameters and Edi signals, whereas, in CPAP/biphasic group, they were getting prefixed positive pressure which may or may not be appropriate to cause optimal inspiratory muscle unloading. Beck et al. [28] in a systematic review on the use of NAVA in children reported the mean Edi values ranging between 8 and 20 mcV. In the present study, the values of Edi peak on NIV NAVA and CPAP are similar to as reported by different authors [21, 24], and falls within the range described by Beck et al. It shows reproducibility and may indicate optimal respiratory muscle unloading on NIV NAVA.

Even though the average MAP (6.5 mmHg) in NIV NAVA was almost the same as in the biphasic/CPAP (6.3/5 mmHg) group in our study, it is difficult to predict the reasons for the increase in work of breathing, and the higher Edi peak on CPAP/biphasic. Variable and inconsistent positive pressure delivery due to intermittent leakage through interface [8, 9], small tidal volume [8], and the agitation of the patient leading to impair detection of the patients breathing efforts could be few reasons. The biphasic mode is comparable to CPAP in infants as it did not prove any advantage over CPAP [29], as they both lack synchronization. Patient-ventilator synchronization is critical to reduce the work of breathing and to achieve successful noninvasive ventilation [29]. Ducharme-Crevier et al. [20] in a prospective cross-over study comparing NIV NAVA to the conventional mode of noninvasive ventilation (CPAP, pressure support ventilation, PSV, and pressure control ventilation, PCV) reported overall significantly reduced asynchrony time in NIV NAVA. Inspiratory trigger dyssynchrony, cycling-off dyssynchrony, and wasted efforts were also significantly reduced in NIV-NAVA. Since NIV NAVA is neurally triggered compared to pneumatic triggered CPAP and biphasic mode, asynchrony index influenced by leaks is insignificant with NIV NAVA [30]. In a study involving the feasibility of implementing NAVA and NIV NAVA in low birth-weight premature infants (mean weight 976 g, range 634-1325 g), Beck et al. [13] showed synchrony comparable to NAVA in NIV NAVA, despite huge leaks. Although there was no significant difference between the two groups in Edi min values in our study, the Edi min values were slightly higher in the CPAP/biphasic group. This could be because of a higher diaphragmatic tone at the end of expiration to maintain the required FRC. 

 As reported in previous studies [21, 23], all infants in our study tolerated the Edi catheter placement. There were no major adverse events during the study. The Edi signals were continuous and consistent, and there was no deterioration of signals with position changes or tube feeding. In all cases, nurses were able to insert and fixate the Edi catheters by themselves with the help of the Edi waveform and electrode positing window.

Limitations of the study are small sample size, and overall fewer infants on biphasic or CPAP alone (six and four), as infants were selected to receive either by the primary care team. The comparison was acceptable since each infant was its own control in the study. The total cumulative time of monitoring was 240 h, and each infant was monitored for a longer duration as compared to some similar previous studies [20-21, 26]. The unavailability of asynchrony index (AI) data in the present study was also a limitation. The AI data could have supported our assumption of higher Edi value on CPAP/biphasic because of poor or ineffective synchrony, but different authors have already shown this as described earlier. Since this study was a pilot study designed for looking at adverse events for a very short period, long-term outcomes such as broncopulmonary dysplasia (BPD) or neurological outcome was not studied.

Our experience in implementing NIV NAVA was straightforward as our unit was already using NAVA for intubated preterm infants. However, NIV NAVA is a comparatively newer mode and requires more experience and practice to set or control as compared to other NIV. It requires more feedback and coordination between bedside nurses, respiratory therapists, and the primary clinical team.

## Conclusions

This study adds to the growing evidence that NIV NAVA can be considered as an acceptable alternative with no significant increase in adverse events compared to the CPAP/biphasic mode. However, NIV NAVA has an added advantage of providing live Edi feedback which may help the clinician in optimizing the lung and respiratory muscle recruitment and offloading. Large-scale randomized control trial (RCT) and studies of long-term prognosis, looking at the incidence of BPD, retinopathy of prematurity (ROP), and periventricular leucomalacia (PVL) are needed.
